# Synergistic Induction of Macrophage Inflammatory Protein-3*α*/CCL20 Production by Interleukin-17A and Tumor Necrosis Factor-*α *in Nasal Polyp Fibroblasts

**DOI:** 10.1097/WOX.0b013e3181bdd219

**Published:** 2009-10-15

**Authors:** Manabu Nonaka, Nozomu Ogihara, Akira Fukumoto, Atsuko Sakanushi, Kaoru Kusama, Ruby Pawankar, Toshiaki Yagi

**Affiliations:** 1 Department of Otolaryngology, Nippon Medical School, Sendagi, Bunkyo-ku, Tokyo, Japan

**Keywords:** CCR6 ligand, chronic sinusitis, MIP-3*α*/CCL20, IL-17 family members

## Abstract

**Background:**

Accumulation of T cells and immature dendritic cells (DCs) is one of the characteristic features of nasal polyps. However, the question remains why these cells accumulate in nasal polyp tissue. Macrophage inflammatory protein-3*α *(MIP-3*α*/CCL20) is a chemokine involved in the migration of T cells and immature DCs into inflammatory tissue sites. Fibroblasts are a rich source of cytokines and chemokines. The objective of this study was to demonstrate the expression of MIP-3*α*/CCL20 in nasal polyp fibroblasts after stimulation with proinflammatory cytokines such as interleukin-17 (IL-17) and tumor necrosis factor-*α *(TNF-*α*).

**Methods:**

Fibroblast lines were established from nasal polyps. MIP-3*α*/CCL20 mRNA expression was evaluated by real-time reverse transcription-polymerase chain reaction (real-time RT-PCR). The amount of MIP-3*α*/CCL20 in the supernatants was measured by enzyme-linked immunosorbent assay (ELISA).

**Results:**

IL-17A and TNF-*α *synergistically induced MIP-3*α*/CCL20 production by nasal polyp fibroblasts in a dose- and time-dependent manner. This synergy was observed by stimulation with TNF-*α *plus IL-17A or IL-17F, but not IL-17E.

**Conclusions:**

Nasal polyp fibroblasts, by producing MIP-3*α*/CCL20, may play an important role in the recruitment of T cells and DCs in upper airway inflammatory lesions such as nasal polyps.

## 

Chronic sinusitis is characterized by persistent inflammation of the sinus, with hyperplasia of the sinus-lining cells that interact with blood-derived cells [1]. Chronic sinusitis often accompanies nasal polyps. Accordingly, the sinus mucosae and nasal polyps demonstrate histologic features that are similar to those in patients with chronic sinusitis. Many of the features of the chronic sinusitis environment, such as the presence of T and B cells, eosinophils, neutrophils and dendritic cells (DCs) in the sinus mucosa and in nasal polyps, suggest a role for chemokines. Chemokines could be released by any of several cell types present in the mucosa, including structural cells such as epithelial cells and fibroblasts and hemopoietic cells such as T cells and eosinophils. Recent advances in sinusitis research have revealed the presence of the following: IL-8, which attracts neutrophils; MCP-4, which attracts eosinophils; and RANTES, which recruits T cells, monocytes, and eosinophils [1,2].

The development of chronic sinusitis is known to be a complex, multifactor process characterized by inflammation of the sinus mucosa. For example, the sinus mucosa and nasal polyps are associated with atopy [3,4]. It was reported that, in the case of nonatopic chronic sinusitis, Th-1 cells are dominant in the ethmoidal mucosa, whereas T_H_2 cells are dominant in atopic chronic sinusitis [4]. Recently, it was reported that Th17 cells infiltrate nasal polyps [5]. However, the mechanism of this migration by Th17 cells remains unclear.

Macrophage inflammatory protein-3*α *(MIP-3*α*)/CCL20 is a CC chemokine identified through bioinformatics, and it is a unique functional ligand for chemokine receptor-6 (CCR6). This receptor is selectively expressed on T cells, immature DCs, and B cells [6-8]. Recently, it was shown that CCR6 is a characteristic of human interleukin-17 (IL-17)-producing T cells [9]. Because MIP-3*α*/CCL20 is expressed in airway diseases,[10] it is possible that MIP-3*α*/CCL20 functions as a chemotactic factor in the migration of Th17 cells and DCs into the sinus mucosa and nasal polyps in patients with chronic sinusitis.

To address this issue, we investigated whether cultured nasal polyp fibroblasts produce MIP-3*α*/CCL20 in response to IL-17A and tumor necrosis factor-*α *(TNF-*α*) (a proinflammatory cytokine) expressed in the sinus mucosa and nasal polyps in chronic sinusitis patients, and also examined 2 other IL-17 family members (IL-17E and IL-17F) for possible effects.

## Methods

### Reagents

Human recombinant IL-17A, IL-17E, IL-17F, IL-4, and TNF-*α *were purchased from R&D Systems, Inc. (Minneapolis).

### Cell Source and Culture

Primary fibroblast lines were established from human polyp biopsy tissues (n = 5) removed at polypectomy, and they were characterized as previously described [11]. Only fibroblast lines between the fourth and sixth passages were used in this study. All the nasal polyp specimens had been obtained from patients with chronic sinusitis (5 males, aged 45.4 ± 18.6 years (mean ± SD)). Two of the patients were atopic, diagnosed on the basis of elevation of at least 1 of the capsulated hydrophobic carrier polymer-radioallergosorbent tests (CAP-RASTs) against 8 common aeroallergens. Each had a cedar pollen score of 2 or more. One of them had a house dust CAP-RAST score of 2. One of the atopic patients had asthma. All subjects had given written informed consent, and the study protocol was approved by the Ethics Committee of Nippon Medical School Hospital.

Before the cytokine assay, fibroblasts (1 × 10^4 ^cells) were plated in 24-well tissue culture plates (Becton Dickinson, Franklin Lakes, NJ). Before real-time polymerase chain reaction (PCR), fibroblasts (2 × 10^6 ^cells) were plated in 60-mm culture dishes (Corning, NY). Plated fibroblasts were allowed to grow to confluence in regular growth medium (Dulbecco's modified Eagle medium) (Invitrogen, Carlsbad, CA) containing 10% fetal bovine serum (Invitrogen), penicillin at 100 units/mL, streptomycin at 100 *μ*g/mL, and amphotericin B at 2.5 *μ*g/mL. The same culture medium was used when fibroblasts were exposed to various stimuli. At specified time points, supernatants were collected and centrifuged for cytokine protein assay, and cells were collected for RNA extraction.

### Analysis of MIP-3*α*/CCL20 mRNA by Quantitative Real-Time Reverse Transcription-Polymerase Chain Reaction

Total cellular RNA was extracted and purified using an RNeasy Mini Kit (Qiagen, Valencia, CA). The total RNA, 2 *μ*g, was reverse-transcribed at 37°C for 60 minutes using random primers (Takara Biochemicals, Tokyo) and Omniscript reverse transcription (Qiagen) according to the manufacturers' protocols.

Quantitative real-time RT-PCR was carried out using the TaqMan assay and ABI Prism 7700 Sequence Detection System (Applied Biosystems, Foster City, CA). The primers and fluorogenic probes for MIP-3*α*/CCL20 and *β*-actin were purchased from Applied Biosystems. The amplification conditions were 2 minutes at 50°C, 10 minutes at 95°C, and 40 cycles of 15 seconds at 95°C and 60 seconds at 60°C.

Data analysis was performed using sequence detector system software (Applied Biosystems). Threshold cycles were used to calculate arbitrary mRNA concentrations by the relative standard curve method. The standard curve was constructed using serial dilutions of cDNA containing the message for MIP-3*α*/CCL20. The level of MIP-3*α*/CCL20 mRNA was normalized against the level of *β*-actin mRNA.

### Cytokine Assay

The levels of MIP-3*α*/CCL20 in culture supernatants were measured by an enzyme-linked immunosorbent assay (ELISA) using a commercially available human MIP-3*α*/CCL20 ELISA kit (R&D Systems). The sensitivity of this assay system was more than 7.8 pg/mL. The data are presented as nanograms of MIP-3*α*/CCL20 per 1 × 10^6 ^cells.

### Statistical Analysis

The paired Wilcoxon test was used for statistical analysis. A *P *value of < 0.05 was considered statistically significant.

## Results

### Effect of IL-17 and TNF-*α *on MIP-3*α*/CCL20 Production by Nasal Polyp Fibroblasts

**Figure 1:**
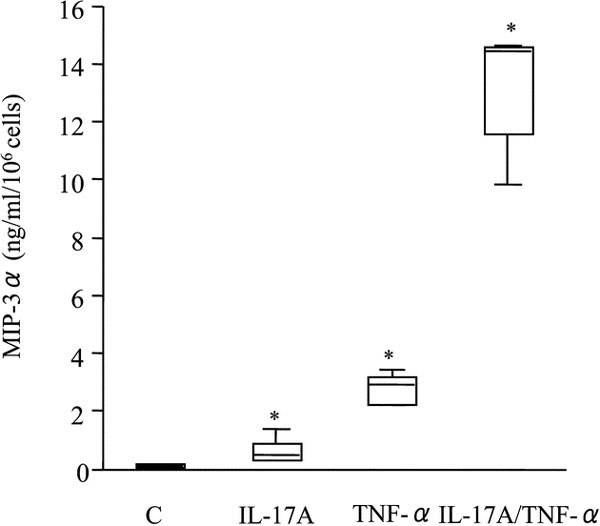
**Effects of IL-17A and TNF-*α *on MIP-3*α*/CCL20 release by nasal polyp fibroblasts**. Cells were cultured with IL-17A (10 ng/mL) and TNF-*α *(10 ng/mL), alone and in combination, and also without stimulation with either, for 72 hours. The MIP-3*α*/CCL20 concentration in the culture supernatant was measured by ELISA. Box plots show the median values for the 25% and 75% interquartiles; the error bars represent the 10th and 90th percentiles (n = 5). *, *P *< 0.05 compared with unstimulated cells. Abbreviation: C, control.

**Figure 2:**
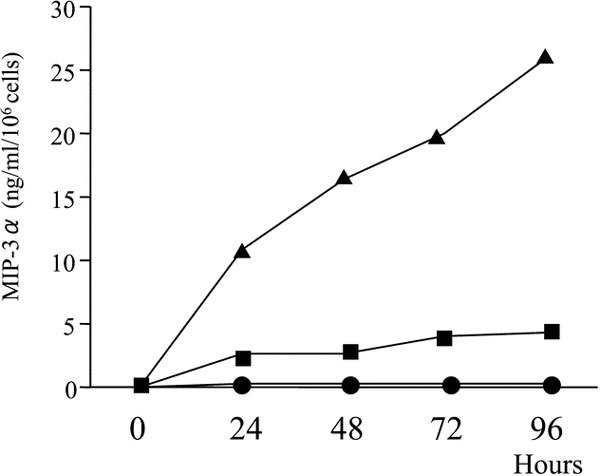
**Kinetics of MIP-3*α*/CCL20 accumulation in supernatants of nasal polyp fibroblast cultures**. Cells were stimulated with IL-17A (10 ng/mL) (●), TNF-*α *(10 ng/mL) (■), or IL-17A and TNF-*α *(▲). Supernatants were assayed for MIP-3*α*/CCL20 after 24, 48, 72, and 96 hours of culture. Results shown are from the average of 2 experiments.

**Figure 3:**
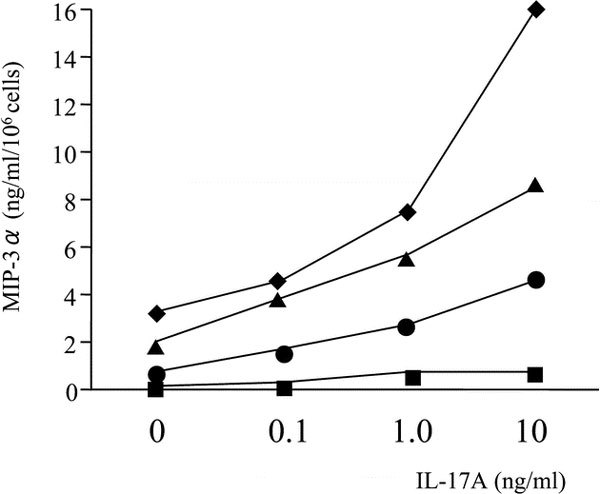
**Dose-effect curves for IL-17A and TNF-*α *on MIP-3*α*/CCL20 production by nasal polyp fibroblasts**. Cells were stimulated with a series of concentrations of IL-17A and a series of TNF-*α *concentrations (■, 0 ng/mL; ●, 0.1 ng/mL; ▲, 1.0 ng/mL; ♦, 10 ng/mL) for 72 hours. Results shown are from the average of 2 experiments.

Nasal polyp fibroblasts were cultured alone or with IL-17A (10 ng/mL) and TNF-*α *(10 ng/mL), alone and in combination. Negligible MIP-3*α*/CCL20 [0.11 ± 0.02 (ng · mL^-1^)/10^6 ^cells; mean ± SEM] was produced in the absence of IL-17A and TNF-*α*. Production of MIP-3*α*/CCL20 [0.65 ± 0.19 (ng · mL^-1^)/10^6 ^cells] was weak in the presence of IL-17A, whereas TNF-*α *induced moderate MIP-3*α*/CCL20 production [2.80 ± 0.23 (ng mL^-1^)/10^6 ^cells; Figure 1]. The combination of IL-17A and TNF-*α *showed synergistic induction of MIP-3*α*/CCL20 production by the nasal polyp fibroblasts [13.14 ± 0.94 (ng mL^-1^)/10^6 ^cells; Figure 1]. The release of MIP-3*α*/CCL20 by the nasal polyp fibroblasts occurred in a dose- and time-dependent manner. In time-course experiments, the MIP-3*α*/CCL20 concentration increased steadily over time, and the synergy between IL-17A and TNF-*α *was observed throughout the experiment (Figure 2). The above-described synergy between IL-17A and TNF-*α *was demonstrated using 10 ng/mL of IL-17A and 10 ng/mL of TNF-*α*. We then tested a series of various combinations of concentrations of IL-17A and TNF-*α*. The synergy between IL-17A and TNF-*α *became more prominent with each increase in the concentration of both IL-17A and TNF-*α *(Figure 3).

**Figure 4:**
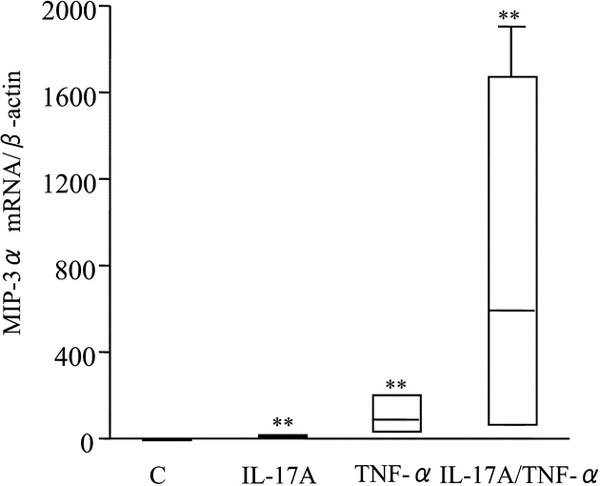
**Effects of IL-17A and TNF-*α *on MIP-3*α*/CCL20 mRNA expression by nasal polyp fibroblasts**. Cells were stimulated with IL-17A (10 ng/mL) and TNF-*α *(10 ng/mL), alone and in combination, for 10 hours, and real-time PCR was performed. Box plots show the median values for the 25% and 75% interquartiles from 3 donors, each studied in triplicate; the error bars represent the 10th and 90th percentiles. The results are normalized against *β*-actin expression. **, *P *< 0.01 compared with unstimulated cells. Abbreviation: C, control.

Real-time PCR was performed for quantitation of the MIP-3*α*/CCL20 mRNA levels. The expression of MIP-3*α*/CCL20 mRNA was weak or undetectable in the unstimulated cells. Upon incubation with IL-17A or TNF-*α*, MIP-3*α*/CCL20 mRNA expression was upregulated after 10 hours of stimulation. Combined stimulation with IL-17A and TNF-*α *induced further upregulation of MIP-3*α*/CCL20 expression in the nasal polyp fibroblasts (Figure 4).

### Comparison of Synergistic Effects of IL-17 Family Members (A, E, and F) and TNF-*α *on Nasal Polyp Fibroblasts

**Figure 5:**
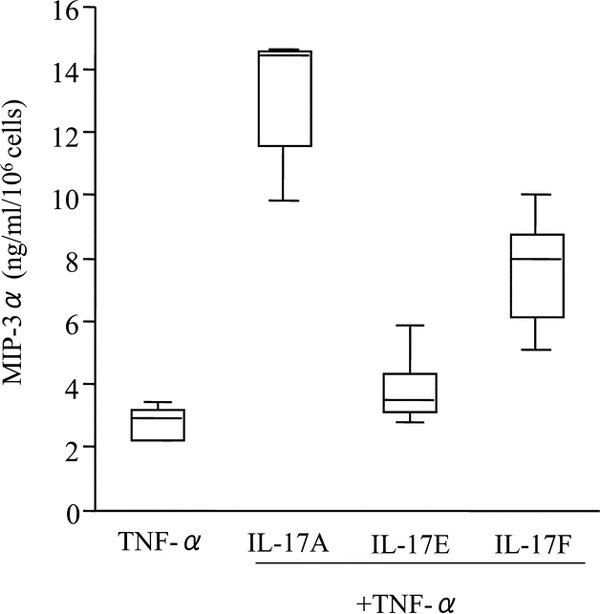
**Effects of IL-17 family members (A, E, and F) and TNF-*α *on MIP-3*α*/CCL20 release by nasal polyp fibroblasts**. Cells were stimulated with IL-17 family members (A or E or F) and TNF-*α*, alone and in combination, for 72 hours. Box plots show the median values with 25% and 75% interquartiles; the error bars represent the 10th and 90th percentiles (n = 5).

We next examined the synergistic effects of several IL-17 family members and TNF-*α *on nasal polyp fibroblasts. Combined stimulation with TNF-*α *plus IL-17A or IL-17F, but not IL-17E, synergistically induced release of MIP-3*α*/CCL20 by the nasal polyp fibroblasts. Figure 5 shows that 72-hour stimulation with IL-17A and TNF-*α *induced about 5 times as much MIP-3*α*/CCL20 [13.14 ± 0.94 (ng · mL^-1^)/10^6 ^cells; mean ± SEM] as that released by TNF-*α *alone [2.80 ± 0.23 (ng mL^-1^)/10^6 ^cells], and costimulation with IL-17F and TNF-*α *induced about 2 to 3 times as much MIP-3*α*/CCL20 [7.59 ± 0.83 (ng mL^-1^)/10^6 ^cells] as that released by TNF-*α *alone. The increase in the release of MIP-3*α*/CCL20 was statistically significant only with IL-17A (Figure 1) (data for IL-17E and IL-17F are not shown).

## Discussion

It is known that when naive CD4 T cells recognize an antigen and are activated, they differentiate into 2 subsets having distinct properties of T_H_1 and T_H_2 and they are both heterogeneous populations of effector/memory cells [12]. Heterogeneity is a particularly prominent feature of the effector/memory CD4^+ ^T-cell population, which induces subsets capable of producing polarized patterns of cytokines that serve specialized functions and have profound effects on the quality of the immune response [13]. It has recently been learned that, in a new helper T-cell population having characteristics different from T_H_1 and T_H_2, IL-23, which is structurally and functionally similar to IL-12, is deeply involved in promotion of differentiation into Th-17 cells that specifically produce IL-17 without producing IFN-*γ*, IL-4, or IL-13 and also in induction of inflammation and some immunity to infections [14].

Recent studies have suggested that human heterogeneous populations of effector/memory cells display distinct patterns of chemokine receptor expression, which are responsible for the differences in patterns of migration by effector/memory versus naive cells [15]. CXCR3 and CCR5 are expressed preferentially on human T_H_1 cells. Conversely, CCR4 is expressed preferentially on T_H_2 cells. It is likely that these receptors are components of positive-feedback loops to reinforce type 1 or type 2 responses in tissues because CXCR3 ligands are induced by interferon-*γ*, the signature cytokine of T_H_1 cells, and CCR4 ligands are induced by IL-4, the signature cytokine of T_H_2 cells. Recently, it was reported that CCR6 is a characteristic of human IL-17-producing T cells [9]. CCR6 is expressed on dendritic cells, B cells, and Th17 cells, and it has only 1 chemokine ligand, MIP-3*α*/CCL20, whose only receptor is CCR6. CCR6 has been reported to have roles in host defense and inflammation [16,17]. In humans, CCR6 and/or MIP-3*α*/CCL20 have been implicated in the pathogenesis of rheumatoid arthritis and inflammatory bowel disease, autoimmune disorders in which Th17 cells are thought to play a crucial role [18,19]. It was also reported that CCR6 and/or MIP-3*α*/CCL20 contributed to airway inflammatory diseases. In mouse models of immune response and diseases, CCR6 and/or MIP-3*α*/CCL20 were required in allergic pulmonary inflammation [20] and contributed to ozone-induced airway hyperresponsiveness [21] and the pathogenesis of cigarette smoke-induced pulmonary inflammation [22]. MIP-3*α*/CCL20 levels in bronchoalveolar lavage from patients with allergic airway inflammation significantly increased and correlated with the allergen-driven influx of lymphocytes after segmental allergen challenge [23]. Considering our data, MIP-3*α*/CCL20 may play a role in upper airway diseases such as chronic sinusitis by recruiting Th17 cells and DCs into nasal polyps.

Chronic sinusitis is defined as sinus inflammation persisting for more than 8 to 12 weeks. It is known that the development of chronic sinusitis is a complex multifactor process characterized by inflammation of the nasal and sinus mucosae. Many studies have shown that the inflammatory cells in chronic sinusitis consist of T and B lymphocytes, DCs, and eosinophils [24]. Nasal polyps are benign outgrowths of the mucosa in the nasal and paranasal cavities, and they can accompany chronic sinusitis. When nasal polyps accompanying chronic sinusitis were classified on the basis of being atopic or nonatopic (determined by allergy skin testing) and their cytokine profiles were analyzed, it was found that IFN-*γ *expression was increased in the nonatopic nasal polyps, whereas IL-4 and IL-5 expressions were increased in the atopic nasal polyps [3]. However, increased TNF-*α *expression was observed in both atopic and nonatopic nasal polyps [3]. Elhini et al [4] similarly classified the ethmoidal mucosa in chronic sinusitis into nonatopic and atopic types (determined by the RAST and the total serum IgE level), and the predominant helper T-cell subsets were investigated. The results showed that the nonatopic ethmoidal mucosa had more cells expressing CCR5 (T_H_1 cells), whereas the atopic ethmoidal mucosa had more cells expressing CCR4 (T_H_2 cells). Furthermore, the number of CCR4^+ ^cells showed a positive correlation with the total serum IgE level, whereas the number of CCR5^+ ^cells showed a negative correlation with the total serum IgE level [4]. It was recently reported that expression of IL-17 is increased in nasal polyps [5]. In addition, that same study found that the IL-17-producing cells in the nasal polyps consisted of 43.3% T lymphocytes [5]. Accordingly, even in chronic sinusitis, it can be surmised that there are 3 different types of inflammation, that is, T_H_1 type, T_H_2 type, and Th17-type. The mechanisms underlying the infiltrations by T_H_1 cells and T_H_2 cells have been investigated for many years, but to date study of the mechanism underlying Th17 cell infiltration has been inadequate.

However, in the present study, we have demonstrated that IL-17A and TNF-*α *synergistically induced MIP-3*α*/CCL20 in nasal polyp fibroblasts. This induction occurred in a dose- and time-dependent manner. Although epithelial cells are thought to be the primary source of MIP-3*α*/CCL20,[25] this study showed that nasal polyp fibroblasts are another source of this chemokine. When IL-23 is expressed at mucosal sites, it has the ability to differentiate Th cells into Th17 cells [14]. It is interesting to speculate whether IL-17A, which is released from Th17 cells, is capable of inducing MIP-3*α*/CCL20 production in nasal polyp fibroblasts synergistically with TNF-*α*, which is abundant in nasal polyps. MIP-3*α*/CCL20, which is released locally, may in turn induce Th17 cells and DCs to migrate out of the vasculature into sites of inflammation via CCR6. This MIP-3*α*/CCL20/CCR6 positive-feedback loop involving Th17 cells, DCs, and fibroblasts amplifies the IL-17 inflammatory response in nasal polyps. The MIP-3/CCL20/CCR6 loop would be analogous to loops involving CXCR3 and its ligands and CCR4 and its ligands for type 1 and type 2 inflammation, respectively.

IL-17A was cloned first, and 6 other IL-17 family members (IL-17A, -17B, -17C, -17D, -17E, and -17F) have subsequently been described. Recently, Ishigame et al [26] reported differential roles for IL-17A and IL-17F in host immune and defense mechanisms. In host defense against mucoepithelial infection, IL-17A was produced mainly by T cells, whereas IL-17F was produced by T cells, innate immune cells, and epithelial cells. Both cytokines activated epithelial innate immune responses, and IL-17A, but not IL-17F, efficiently induced cytokines in macrophages [26]. Among the IL-17 family members, the IL-17F isoforms 1 and 2 (ML-1) have the highest degree of homology with IL-17A (55% and 40%, respectively), followed by IL-17B (29%), IL-17D (25%), IL-17C (23%), and finally IL-17E, the most distant (17%) [27]. Because IL-17F has a high degree of homology with IL-17A and has a similar spectrum of activity in terms of induction of G-CSF and CXCL8, perhaps IL-17F also uses IL-17R, a receptor for IL-17A, for signaling. Considering that IL-17E uses IL-17RB, one of the other receptors for IL-17 family members, IL-17R may be necessary for the synergistic induction of MIP-3*α*/CCL20 by IL-17A and TNF-*α *and by IL-17F and TNF-*α*. Further investigations are needed to clarify the mechanism(s). Increasing evidence shows that IL-17 family members play active roles in inflammatory diseases, autoimmune diseases, and cancer. Among the IL-17 family members, expression of IL-17A, -17E, and -17F has been documented in lower airway diseases [10,28,29]. Our results suggest that IL-17A and IL-17F, but not IL-17E, contribute to infiltration of Th17 cells and DCs into upper airway inflammatory sites such as nasal polyps through the production of MIP-3*α*/CCL20 by fibroblasts.
